# Challenges in the Assembly and Handling of Thin Film Capped MEMS Devices

**DOI:** 10.3390/s100403989

**Published:** 2010-04-20

**Authors:** Jeroen J. M. Zaal, Willem D. van Driel, G.Q. Zhang

**Affiliations:** Delft University of Technology, Department PME, Mekelweg 2, 2628 CD Delft, The Netherlands; E-Mails: Willem.van.Driel@Philips.com (W.D.v.D.); G.Q.Zhang@TUDelft.nl (G.Q.Z.)

**Keywords:** MEMS, wafer level thin film package, packaging, resonator

## Abstract

This paper discusses the assembly challenges considering the design and manufacturability of a Wafer Level Thin Film Package in MEMS applications. The assembly processes are discussed. The loads associated with these processes are illustrated and evaluated. Numerical calculations are combined with experimental observations in order to estimate the assembly risks. Our results emphasize the need for concurrent design for assembly.

## 1. Introduction

With the rising interest in Micro Electrical Mechanical Systems (MEMS) due to expanding application areas and new products opportunities the need for reliable and cost-effective MEMS development is becoming more and more important. One of the most important differences between MEMS and traditional IC products are the (possible) use of moving parts. These can include: cantilevers, membranes and resonators. Cantilevers can be used in switching applications [1] or data storage applications. Membranes find applications in microfluidics and microphone applications. Finally, resonators [2] can be used as sensors for temperature [3], gas [5], particles [6], motion [7], pressure [8] and in many other applications such as oscillators [9,10] or several medical tasks [4].

Although our research merely focuses on MEMS resonators using a Wafer Level Thin Film Package (WLTFP) the described problems and risks apply to all WLTFP’s in general. MEMS resonators have tremendous advantages over conventional resonators, because they are small, and can easily be batch processed. When combining the resonator with a WLTFP, a very thin product can be produced. The processing of a whole wafer in one go means that a large number of resonators can be packaged at once. This results in a very low risk of contamination since the unsealed resonator does not leave the cleanroom. After the sealed resonator structure is created, the resonator can be used in a multi chip module and serve as an oscillator.

Besides these advantages, one of the main drawbacks is the fragile nature of the WLTFP. This can cause many problems during fabrication and/or lifetime testing of these devices. The fragile nature and packaging influences on MEMS in general is reported to be a roadblock in fast and effective MEMS development [11,12].

This paper discusses the assembly challenges considering the design and manufacturability of a WLTFP in MEMS applications. Section 2 will briefly introduce the concept of WLTFP’s, followed by Section 3 and the respective subsections treating the relevant assembly steps: wafer thinning, wafer dicing, wire bonding, and plastic encapsulation.

## 2. Wafer Level Thin Film Package Design

**Figure 1.**
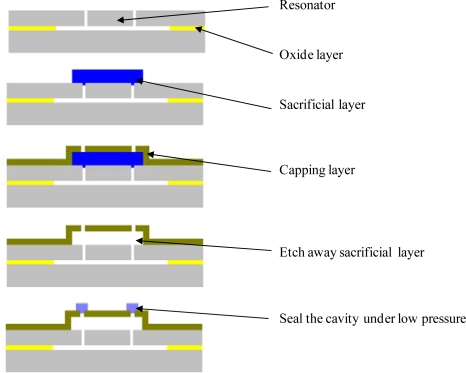
An example of a production method for a WLTFP.

During the design and optimization of a WLTFP, the loads occurring during both the assembly processes and the lifetime should be considered in order to increase the reliability and manufacturability. Choice of material is an important factor in this process since material properties as brittleness, plasticity and microstructure influence both the mechanical strength and the hermiticity of the WLTFP. Figure 1 provides an example production process of a WLTFP. These types of processes are under constant development for different type of process flows and temperature ranges and are described in several papers [13–16].

Loads introduced during the production process, such as stresses due to thermal mismatch, influence the yield during assembly and lifetime reliability. A WLTFP that experiences a low intrinsic stress level before assembly is more robust since it can cope with more stress due to handling and assembly. This requires investigations into the stress levels induced by the assembly process in order to design a stronger and more reliable cavity.

## 3. Wafer Level Thin Film Package Assembly

**Figure 2.**
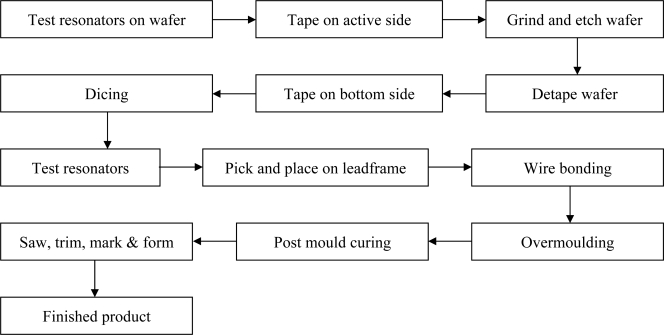
Production flow for plastic encapsulation of a plastic overmoulded package.

After production, assembly is the next step for the resonator. This research focuses on packaging resonators in plastic moulded packages. A typical assembly flow is depicted in Figure 2.

Each step in the assembly process imposes different types of mechanical loading on the WLTFP. Loading mechanisms that occur during these steps are discussed in the following subsections.

### 3.1. Wafer Thinning

**Figure 3.**
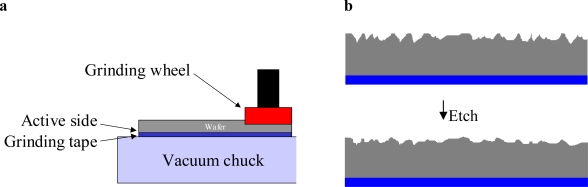
(a) Wafer thinning by grinding; (b) Etching away of crack initiation points in order to increase the wafer strength [17].

Wafer thinning is composed of three steps: applying the grinding tape, thinning the wafer and removing the grinding tape. The grinding tape is needed to fix the wafer to the vacuum table used to support the wafer. Figure 3 schematically depicts the grinding setup where the bulk of the material is removed by means of a mechanical grinding process. The last step is to etch away the rough peaks created by the grinding abrasive in order to increase the wafer strength [17].

**Figure 4.**
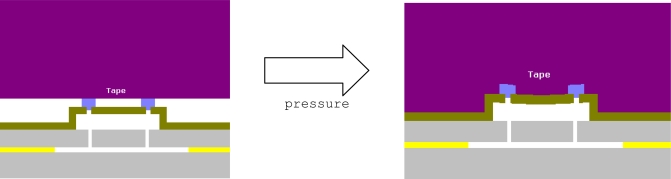
Tape application to the front side of a wafer.

**Figure 5.**
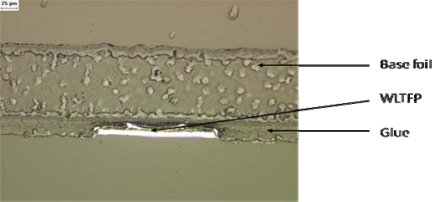
WLTFP embedded into the glue layer on the tape.

The grinding tape application process can damage the WLTFP due to the applied pressure. This pressure is needed to create a sufficient adhesive strength. When tape is applied to the WLTFP side of the wafer, the tape will land on the WLTFP’s itself, which are sticking out above the surface. The application pressure will then force the tape to contact the remaining wafer surface. In other words the tape will eventually embed the WLTFP structure. Figure 4 visualizes this loading mechanism and while Figure 5 shows an embedded WLTPF in a cross-section of the tape.

**Figure 6.**
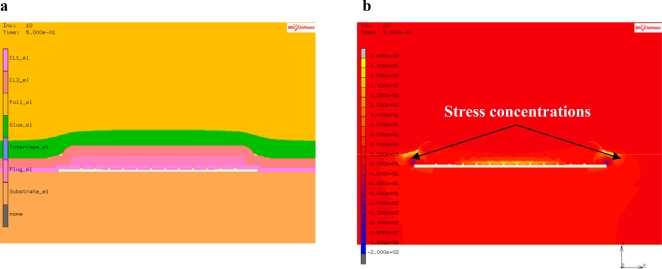
(a) Tape application deformations; (b) Stress distribution during tape application.

3D and 2D Finite Element (FE) simulations are performed to calculate the stresses in the WLTFP during tape application. The resulting deformations and stress levels of a WLTFP during tape application be found in Figure 6. The yellow layer in Figure 6(a) is the base film of the tape and the green layer is the glue. This is forced around the WLTFP by the applied pressure but the glue does not have full contact everywhere. Contact algorithms are used to mimic the embedding of the WLTFP in the glue. Figure 6(b) depicts the maximum principal stress corresponding with this loading mechanism. The edges and the center of the cavity are loaded due to the bending in the freestanding WLTFP layers. The maximum principal stress due to the application of the tape is 180 MPa in this model with a WLTFP span of 100 μm.

**Figure 7.**
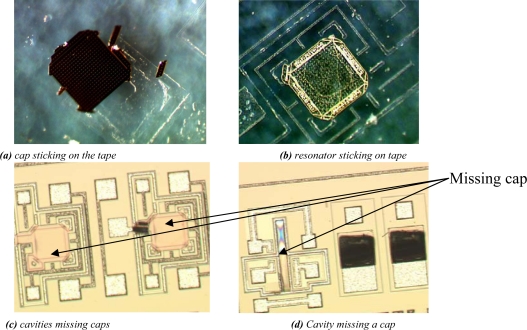
Damage due to tape application and removal.

The (2D) model contains two materials for the grinding tape: the foil (E = 50 MPa, ν = 0.3) and the glue (E = 20 MPa, ν = 0.3). The WLTFP contains several layers of silicon nitride (E = 190 GPa, ν = 0.2) and aluminium (E = 60 GPa, ν = 0.3). The application of the tape is done by means of an edge load. The tape removal process on the active side is hazardous in the sense that the release glue will apply tensile forces on the WLTFP caps and plugs. Damage to WLTFP’s due to tape application and removal on are depicted in Figure 7. This specific wafer did not undergo any grinding process between tape application and removal. This means all damage is caused by either the applying of the tape or the tape removal forces.

**Figure 8.**
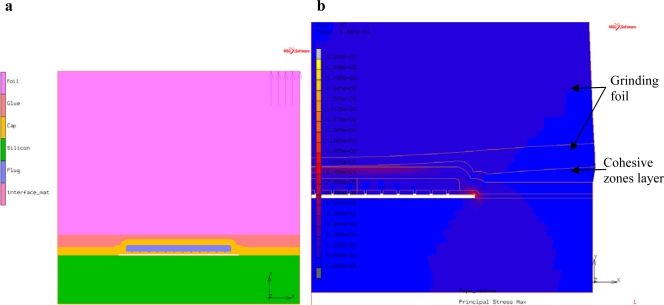
(a) Tape removal model; (b) Principal stressed caused by tape removal.

Simulation of the tape removal process can identify the regions of the WLTFP that experience the highest stress concentrations. These regions should then be stiffened to increase the design robustness. To simulate the tape removal process, a model using the cohesive zones technique [18] is made by modifying the geometry from the earlier presented simulations. The modified model is depicted in Figure 8(a). The geometry depicted in Figure 8(a) is a 2D representation of a 100 μm wide cavity with a matrix of plugs sealing the cavity.

The stress distribution in Figure 8(b) shows that the stress concentrations are found in the region near the corners of the span and the middle part of the freestanding layer. The maximum principal stress is around 200 MPa for this specific type of tape and cavity combination.

### 3.2. Wafer Dicing

**Figure 9.**
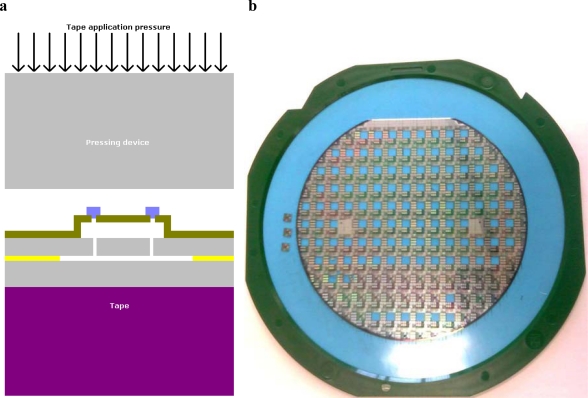
(a) Loading mechanism during dicing tape application; (b) Diced wafer on dicing tape.

The dicing process consists of a tape application step and a dicing step. The tape is applied to the backside of the wafer, instead of the front side that was used during the wafer thinning. The wafer once more undergoes pressure loading when the tape is applied to the backside. Careful support to the WLTFP side of wafer is needed since the soft tape is on the back side. No soft tape is present to protect the WLTFP from pressure exerted to stick the wafer to the foil. This is illustrated in Figure 9(a). An example of a wafer on dicing tape is depicted in Figure 9(b).

**Figure 10.**
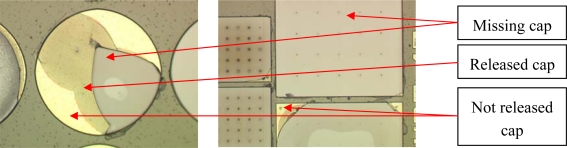
Cracked wafer level thin film packages during wafer dicing.

Examples of damages to WLTFP’s during the wafer dicing process are shown in Figure 10. The yellow color is the stack of sacrificial material and capping material, the brown color is freestanding capping material and the grey is the bare silicon without any layers on top.

**Figure 11.**
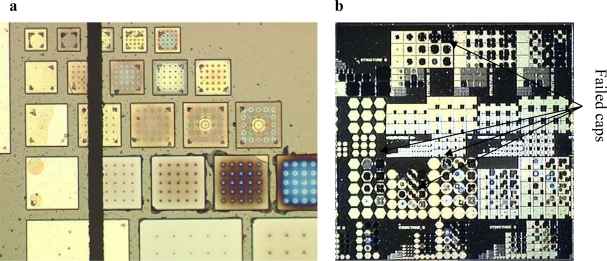
(a) The saw lane shows little damage; (b) Caps fail on this chip with no specific location.

As shown in Figure 11(a) the dicing blade caused some minor damage. It is observed that the damage level is approximately equal over the 10 × 10 mm^2^ die surface in Figure 11(b). Since the damage is found all over the wafer instead of only next to the dicing lane it can be concluded that the damage is not caused by the dicing blade. Instead, the damage is ascribed to the wafer handling.

When using a laser dicing process instead of diamond blade dicing one of the main concerns is the debris around the sawing lanes that needs to be removed. New methods with little or no debris are in development to make laser dicing an attractive alternative for (thin) MEMS wafers [19].

### 3.3. Die Attachment

During the placement of the die on the leadframe, the die needs to be removed from the tape by means of a pick and place operation. The same holds for a flip-chip application. The cavity can be damaged by the gripper when it is touched. On the contrary the vacuum used to hold the chip will not damage low pressure cavities since the differential pressure only decreases due to the very low cavity pressure. The pick and place force is in the order of 1N [20], this corresponds to a 1MPa suction force on the chip when a vacuum collet is used with a 1 mm^2^ internal cross-section. These forces are not significant in comparison to other process induced stresses.

### 3.4. Wire Bonding

**Figure 12.**
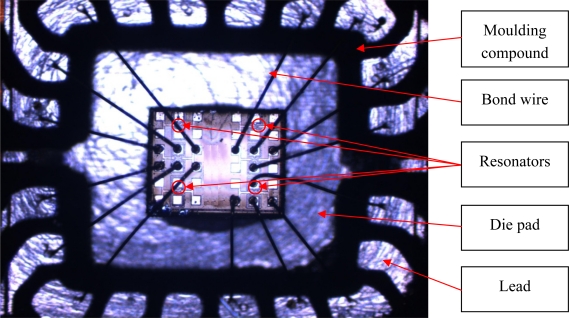
A prototype die-attached and wire bonded chip with four resonator blocks.

The wire bonding process uses a high frequency rubbing action to attach the bondwire to the bondpad. This sinusoidal shaped force is carried into the device by the bondpad construction. The process frequency can act as an actuator on any part of the MEMS. Depending on the geometry and materials of the MEMS under consideration, it is necessary to analyze to likelihood of problems. If one of the eigenfrequencies of the MEMS or the WLTFP is (very) close to the bonding frequency, it may start to resonate. Since the resonance frequency depends on both mass, stiffness and pre-stresses, every case is different. A different stress level in the WLTFP due to annealing can lead to a different effective stiffness and thus solve or create a resonance problem. Figure 12 depicts a die-attached and wire-bonded die with several resonators present.

**Figure 13.**
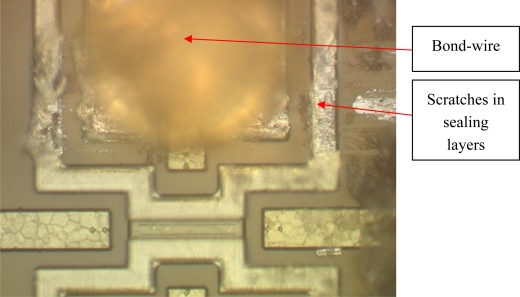
Bond needle damage to a sealing structure.

Besides resonance, the geometry of the MEMS can cause damage during wire bonding. Due to thick layers or high structures the movement of the bond-needle needs to be programmed in 3 directions while usually in plane movements occur. Figure 13 depicts a resonator chip with damage to the sealing layers due to scratching by the bond capillary.

### 3.5. Overmoulding

The WLTFP should protect the MEMS device and there should be no contact between the cap and the device during the overmoulding process. In the overmoulding process pressures of 60–90 bar are used to liquidize the epoxy and overflow the die. Due to the imposed pressure, the cap may bend and thereby close the cavity or even crack. The pressure acting on a WLTFP is not equal for each WLTFP in the mould due to the viscous nature of the epoxy moulding compound. Pressure is needed to flow the epoxy to each corner of the mould. The flow front should, by default, be at atmospheric pressure, meaning that WLTFP just behind the flow front experiences a low pressure. The injection pressure is the pressure measured in the plunger of the injection moulding machine. The real experienced pressure by the WLTFP is thus a function of location in the mould [21]. WLTFP’s closer to the runner will experience a higher pressure than WLTFP’s far away from the runner. When the filling of the cavity is completed, pressure will rise everywhere due to the lack of empty space to fill. However, due to curing the pressure on the WLTFP will not be equal to the injection pressure since the EMC is curing rapidly and reinforcing the cavity. This is caused by the high mould temperature which affects the curing kinetics.

After moulding the significant magnitude of the curing shrinkage of the EMC [22] can load the WLTFP comparable with an external pressure. The curing shrinkage can be 1 to 2 percent in each direction.

**Figure 14.**
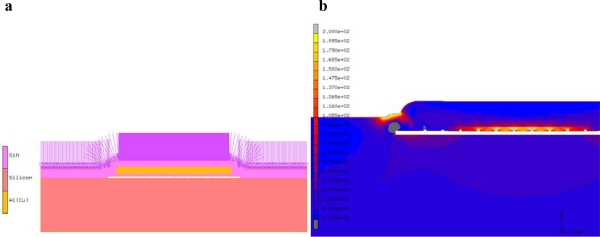
(a) Model of the WLTFP under moulding pressure (arrows represent the moulding pressure); (b) The maximum principal stress in the WLTFP due to an 80 bar moulding pressure.

Simulations of a moulding process should thus include curing kinetics and cure shrinkage in combination with a flow analysis when an accurate estimation of the moulding process loads is needed. An easier approximation can be made by applying the injector pressure to the model. However, this is an overestimation and the cavity should thus be more robust than needed. Figure 14(a) depicts such a model. The model is a modified version from the models earlier described using the same materials and geometry. A pressure of 8 MPa is added as representation of an 80 bar moulding pressure. The maximum stresses found are in the order of 250 MPa. The high stress regions are located near the edge of the cavity, which is a bending point, and on the underside of the cavity near the centre.

## 4. Discussion and Conclusions

Although the possibilities of MEMS products in low-cost packages are large, the challenges with respect to manufacturability and/or yield are significant. This paper illustrates that the assembly processes needed to package a MEMS chip are important during the design of the MEMS chip since they impose significant loads.

**Figure 15.**
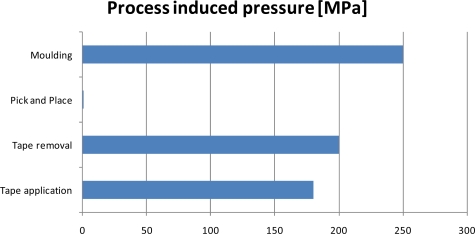
Process induced pressures on the WLTFP.

Possible loading conditions during the most common assembly processes are discussed and illustrated. The process induced stresses are summarized in Figure 15.

Based on experimental observations combined with Figure 15 a ranking of the most hazardous assembly processes for WLTFP’s can be made, being:

1. Wafer thinning. The most hazardous is the wafer thinning process due to the direct contact with the grinding tape and the process pressure used.
2. Dicing. Dicing is a hazardous process due to the application to the tape and the risk of debris and subsequent cleaning damage.
3. Overmoulding. The moulding process is less dangerous than the previous two processes but can cause problems in yield due to the different loading throughout the mould.

A robust and accurate WLTFP design should consider assembly loads and try to avoid stress concentrations after wafer processing. These stress concentrations can be caused by, for example, CTE (Coefficient of Thermal Expansion) mismatches or poor geometry design. In this paper, we have combined numerical calculations with experimental observations in order to estimate the assembly risks. Our results clearly emphasize the need for concurrent design for assembly. Solutions to reduce the stresses in the critical areas are under investigation.
